# Comparison of Hematological and Biochemical Parameters of SARS-CoV-2-Positive and -Negative Neonates of COVID-19 Mothers in a COVID-19 Hospital, Odisha State

**DOI:** 10.7759/cureus.23859

**Published:** 2022-04-05

**Authors:** Santosh K Panda, Subhra Snigdha Panda, Deepti D Pradhan, Manas K Nayak, Arpan Ghosh, Nirmal K Mohakud

**Affiliations:** 1 Neonatology, Kalinga Institute of Medical Sciences, Bhubaneswar, IND; 2 Microbiology, Kalinga Institute of Medical Sciences, Bhubaneswar, IND; 3 Technology, Kalinga Institute of Industrial Technology (KIIT) Technology Business Incubator, Bhubaneswar, IND; 4 Pediatric Medicine, Kalinga Institute of Medical Sciences, Bhubaneswar, IND

**Keywords:** sars-cov-2, biochemical paramenter, hematological, asymptomatic, symptomatic, neonates

## Abstract

Introduction

Data are scarce on the hematological and biochemical changes caused by the severe acute respiratory syndrome coronavirus 2 (SARS-CoV-2) in neonates. This study aimed to compare hematological and biochemical parameters in SARS-CoV-2-positive neonates with healthy neonates born to mothers diagnosed with coronavirus disease 2019 (COVID-19) and assess disease severity in both groups.

Methodology

This prospective observational study was conducted at a COVID-19 hospital at Kalinga Institute of Medical Sciences, Bhubaneswar, Odisha, India, from May 1 to November 30, 2020. Forty-eight babies, including 39 inborn and nine outborn, were enrolled in the study after their parents provided written informed consent. Neonates were diagnosed with COVID-19 via nasopharyngeal real-time reverse transcription-polymerase chain reaction testing. The hematological and biochemical parameters of these 48 neonates were recorded and analyzed.

Results

SARS-CoV-2-infected neonates had lower hemoglobin, neutrophil to lymphocyte ratio, total white blood cell count, and absolute neutrophil count compared to noninfected babies (p<0.05). All SARS-CoV-2-infected neonates had serum transaminase levels and renal function tests within reference ranges. We saw no significant differences in hematological and biochemical parameters among asymptomatic SARS-CoV-2-infected and noninfected neonates.

Conclusions

Hematological and biochemical parameters between asymptomatic SARS-CoV-2-infected and non-infected neonates were similar. The blood count abnormalities found in SARS-CoV-2-infected neonates could be due to other associated neonatal comorbidities. According to our results, asymptomatic SARS-CoV-2-infected newborns need close monitoring rather than a battery of investigation.

## Introduction

The ongoing coronavirus disease 2019 (COVID-19) pandemic caused by infection with the severe acute respiratory syndrome coronavirus 2 (SARS-CoV-2) has led to 491 million cases and 6.17 million deaths as of March, 2022 [1]. SARS-CoV-2 infects patients of all ages, including vulnerable groups like newborns and patients with advanced age. Symptomatic neonates most commonly present with respiratory distress, with fever and feeding intolerance. Data on the impact of SARS-CoV-2 infection in neonates are limited, and data are scarce on neonatal clinical presentations to allow clinicians to diagnose without hematological and biochemical parameters. This makes preventative interventions like isolation and treatments like early initiation of feeding, need for O_2_, and to start respiratory support or not, challenging for neonates. While COVID-19 causes severe manifestation in adults with comorbidities, most children and neonates with COVID-19 have good outcomes [2,3]. A SARS-CoV-2-induced cytokine storm may result in marked lymphocytic apoptosis leading to lymphopenia and leukocytosis in adults, but this process is rare in children [4,5]. As of now, data on hematological parameters in COVID-19-positive neonates and their level of disease severity are scarce. Therefore, this study aimed to evaluate the hematological and biochemical parameters in COVID-19-positive neonates and compare them with baseline hematology parameters of COVID-19-negative neonates born to COVID-19-positive mothers.

## Materials and methods

This prospective observational study was conducted at a COVID-19 hospital at Kalinga Institute of Medical Sciences (KIMS), Bhubaneswar, Odisha, from May 1 to November 30, 2020. Forty-eight babies, including 39 inborn and nine outborn, were enrolled after their parents provided written consent. All babies born out of SARS-CoV-2-positive mothers and referred to this COVID-19 hospital peripheral hospitals during the study period were enrolled. According to the 2020 Indian Council of Medical Research (ICMR) guidelines, mothers were considered SARS-CoV-2 positive if delivery occurred within 10 days of detection of the virus. Single nasopharyngeal (NP) swabs were used for diagnosis by real-time reverse-transcriptase polymerase chain reaction (RT-PCR) as per the ICMR guidelines [2]. Within 24 hours of delivery, all babies born to COVID-19-positive mothers underwent NP swab and RT-PCR tests. We collected blood for complete blood count (CBC) and C reactive protein (CRP) values 12 hours after birth. At 48 hours after birth, we measured liver function test (LFT), renal function tests (RFT), serum electrolytes, calcium, coagulation profile (prothrombin time and activated partial thromboplastin time), and thyroid-stimulating hormone (TSH) for thyroid function screening. CBC evaluation was conducted using a hematology analyzer (DXH-800; Beckman Coulter, Indianapolis, IN, USA). LFT, RFT, electrolytes, and CRP were measured using a VITROS 5600 (OrthoClinical Diagnostics, Raritan, NJ, USA) and a DXC 700 AU clinical chemistry analyzer (Beckman Coulter). We used a Sysmex Automated Blood Coagulation Analyzer CA-500 (Sysmex Corp., Kobe, Hyogo, Japan) and a Trombochord S analyzer to measure the coagulation profile. We used Manroe’s chart for absolute neutrophil count (ANC) for term neonates and Mourinho's chart for ANC in very low birth weight babies.

Asymptomatic neonates were kept with their mothers unless the mother was critically sick. Asymptomatic neonates were allowed to continue breastfeeding as per standard protocols [6]. We collected demographic data, epidemiological features, and clinical presentations from case sheets.

Statistical analysis

We reported descriptive statistics. The laboratory parameters of SARS-CoV-2-positive symptomatic/asymptomatic and negative neonates were compared. We used IBM SPSS Statistics for Windows, Version 20.0 (IBM Corp., Armonk, NY, USA) to analyze the results. We considered p<0.05 as statistically significant. The KIMS Research and Development committee approved the study (KIMS/RD-48/2020/09).

## Results

Forty-eight neonates were included in the study. Of 39 neonates delivered in the COVID-19 hospital, three (7.6%) were COVID-19 positive. Nine outborn neonates with COVID-19 were admitted to the COVID-19 neonatal intensive care unit. Among the 12 neonates with COVID-19, eight had no symptoms, one had severe hypoxic-ischemic encephalopathy (HIE), and three had blood culture positive sepsis (one of whom died due to sepsis with disseminated intravascular coagulation). Of 36 SARS-CoV-2-negative neonates, four were symptomatic (three with HIE, one with blood culture positive sepsis). The demographic profile of all neonates is presented in Table 1.

**Table 1:** Demographic characteristics of neonates (n=48) born from COVID-19-positive mothers ^a^ Mean birth weight: 2.31 ± 0.862 kg; mean gestation: 36.06 ± 3.16 weeks ^b^ Mean birth weight: 2.56 ± 0.708 kg.; mean gestation: 37.41 ± 3.017 weeks. Abbreviations: COVID-19, coronavirus disease 2019; SARS-CoV-2, severe acute respiratory syndrome coronavirus 2

Parameters	SARS-CoV-2-Positive, n (%)^a^	SARS-CoV-2-Negative, n (%)^b^	Total Percentage
Low birth weight	5 (41.6)	11 (30.5)	33.3%
1.5-2.5 kg	2 (16.6)	8 (66.6)	20.83%
1-1.5 kg	1 (8.3)	3 (8.3)	8.3%
<1 kg	2 (16.6)	0	4.1%
Term	8 (66.6)	30 (83.3)	79.1%
Preterm (<37 weeks)	4 (33.3)	6 (16.7)	20.82%
Late preterm (34-36+6) weeks	2 (16.6)	3 (8.3)	10.4%
Very preterm (28–33+6) weeks	2 (16.6)	2 (5.50)	8.3%
Extreme preterm (<28 weeks) weeks	0	1 (2.7)	2%
Male	10 (83.3)	15 (41.6)	52.5%
Female	2 (16.7)	21 (58.4)	47.5%
Vaginal delivery	4 (33.3)	13 (36.1)	35.4%
Cesarean delivery	8 (66.6)	23 (63.9)	64.9%
Death	1 (8.3)	0	2%

Among SARS-CoV-2-positive neonates, two (16.6%) had anemia (i.e., hemoglobin <13g/dL), one (8.3%) had leukocytosis, and one neonate (8.3%) had leucopenia, thrombocytopenia, and blood culture-positive sepsis. Of the two anemic newborns, one was very preterm (29 weeks) with very low birth weight (1100 g), neonatal sepsis, and grade 1 intraventricular hemorrhage. The other was a term neonate with mild anemia (hemoglobin 12.8 g/dL). Two SARS-CoV-2-positive symptomatic (blood culture-positive sepsis) neonates had received fresh frozen plasma for coagulopathy.

In the subgroup analysis of the asymptomatic SARS-CoV-2-positive neonates, none had an abnormal total leukocyte count (TLC), ANC, total platelet count, and absolute lymphocyte count. However, hemoglobin, TLC, ANC, and neutrophil to lymphocyte ratio (NLR) were significantly lower in neonates infected with SARS-CoV-2 (p<0.05) (Table 2). The CBC and coagulation profiles of asymptomatic neonates regardless of SARS-CoV-2 infection had no significant differences (Table 3).

**Table 2:** Comparison of hematological parameters of SARS-CoV-2 positive (n=12) and negative (n=36) neonates Abbreviations: SARS-CoV-2, severe acute respiratory syndrome coronavirus 2; Hb, hemoglobin; PCV, packed cell volume; TLC, total leukocyte count; ANC, absolute neutrophil count; ALC, absolute lymphocyte count; NLR, neutrophil-lymphocyte ratio; TPC, total platelet count; PT, prothrombin time; APTT, activated partial thromboplastin time: Cumm, cubic millimeter

Parameters	SARS-CoV-2 Positive, Mean ± SD	SARS-CoV-2 Negative, Mean ± SD	P-Value
Hb (g/dl)	15.14 ± 2.53	17.26 ± 3.07	0.036
PCV (%)	47.2 ± 8.32	52.81 ± 8.62	0.055
TLC (cells/Cumm)	12695.83 ± 8780.25	18400 ± 7038.58	0.008
ANC (cells/Cumm)	7459.41 ± 7083.93	12387.2 ± 5816.19	0.004
ALC (cells/Cumm)	4020.62 ± 2534.32	4003.4 ± 1339.24	0.625
NLR	2.22 ± 1.95	3.36 ± 1.93	0.027
TPC (cells/Cumm)	2.46 ± 0.83	2.14 ± 0.60	0.211
PT (seconds)	24.24 ± 21.48	18.11 ± 4.75	0.751
APTT (seconds)	52.39 ± 31.35	37.07 ± 6.18	0.006

**Table 3:** Comparison of hematological parameters of asymptomatic SARS-CoV-2 positive (n=8) and negative (n=32) neonates Abbreviations: SARS-CoV-2, severe acute respiratory syndrome coronavirus 2; Hb, hemoglobin; PCV, packed cell volume; TLC, total leukocyte count; ANC, absolute neutrophil count; ALC, absolute lymphocyte count; NLR, neutrophil-lymphocyte ratio; TPC, total platelet count; PT, prothrombin time; APTT, activated partial thromboplastin time; Cumm, cubic millimeter

Parameters	SARS-CoV-2-Positive, Mean ± SD	SARS-CoV-2-Negative, Mean ± SD	P-Value
Hb (g/dl)	15.175 ± 1.973	17.321 ± 2.941	0.05
PCV (%)	47.75 ± 6.95	52.98 ± 8.23	0.10
TLC (cells/Cumm)	15862.5 ± 8992.3	17287.5 ± 5500.07	0.28
ANC (cells/Cumm)	9448.62 ± 7848.62	11314.53 ± 4246.33	0.13
ALC (cells/Cumm)	4855.5 ± 2479.03	4045.37 ± 1349.87	0.55
NLR	2.192 ± 1.62	3.02 ± 1.45	0.16
TPC (cells/Cumm)	2.7 ± 0.777	2.13 ± 0.63	0.07
PT (seconds)	15.15 ± 3.81	18.19 ± 4.61	0.62
APTT (seconds)	35 ± 10.3	37.06 ± 5.96	0.45

All SARS-CoV-2-infected newborns had serum transaminase levels and renal function tests within reference ranges. Two symptomatic SARS-CoV-2-positive newborns had hyponatremia (<130 meq/L), associated with blood culture-positive septic shock. One neonate had early-onset hypocalcemia associated with respiratory distress. All SARS-CoV-2 positive neonates had TSH levels within the reference range. There was no significant difference in CRP, LFT, RFT, serum electrolytes, and TSH between SARS-CoV-2 positive and negative neonates (Table 4). Moreover, there were no significant differences in CRP, LFT, RFT, serum electrolyte, and serum TSH function between asymptomatic SARS-CoV-2-positive neonates and healthy neonates.

**Table 4:** Biochemical parameters of all neonates (n=48). ^a ^Positive CRP (>10 mg/dl): n=4 (33%) ^b ^Positive CRP (>10 mg/dl): n=9 (25%), p=0.71. Abbreviations: CRP, C-reactive protein; AST, aspartate aminotransferase; ALT, alanine aminotransferase; TSH, thyroid stimulating hormone; SARS-CoV-2, severe acute respiratory syndrome coronavirus 2

Parameters	SARS-CoV-2-Positive, Mean ± SD^a^	SARS-CoV-2-Negative, Mean ± SD^b^	P-Value
Mean CRP (mg/dl)	19.09 ± 28.65	9.83 ± 11.35	0.64
Urea (mg/dL)	23.5 ± 10.42	26.9 ± 11.6	0.431
Creatinine (mg/dL)	0.72 ± 0.24	0.75 ± 0.36	0.784
Bilirubin (Total) in mg/dL	8.14 ± 4.54	9.29 ± 4.39	0.567
AST (*units*/*L*.)	38.58 ± 7.78	51.44 ± 87.95	0.61
ALT (*units*/*L*.)	29.4 ± 6.97	40.16 ± 30.65	0.237
Sodium (mEq/L)	134.9 ± 6.03	136.5 ± 6.37	0.45
Potassium (mEq/L)	4.59 ± 0.74	4.5 ± 0.76	0.79
Calcium (mg/dL)	8.84 ± 1.11	8.35 ± 1.21	0.22
TSH (ng/dL)	4.28 ± 2.53	5.53 ± 4.61	0.305

## Discussion

Our study focuses on different aspects of hematological and biochemical parameters in SARS-CoV-2-positive and -negative neonates born to COVID-19-positive mothers. TLC, ANC, and NLR were significantly lower in the SARS-CoV-2-positive newborns than in healthy controls (p<0.05). However, lower TLC and ANC counts among SARS-CoV-2-positive symptomatic newborns may be due to neonatal comorbidities. Moreover, the hematological and biochemical parameters were similar regardless of SARS-CoV-2 infection status among newborns. Therefore, investigation and treatment should be used judiciously in a resource-limited setting.

SARS-CoV-2 infection represents a new lesson in medical science. The immunological response of neonates toward a SARS-CoV-2 infection is still poorly understood. The hematological manifestation of SARS-CoV-2 infection among children differs from adult patients in that neonates and pediatric patients are less symptomatic [7]. Due to overlapping clinical signs and symptoms with bacterial sepsis, an isolated SARS-CoV-2 infection is challenging to identify clinically during the early neonatal period. Of 12 SARS-CoV-2-positive newborns, we found that 33% had sepsis or HIE, but the literature does not describe the etiology of morbidities in SARS-CoV-2-positive neonates. In this study, three of the four symptomatic SARS-CoV-2-positive neonates had sepsis, and three of the SARS-CoV-2-negative neonates had HIE. Zeng et al. reported that among 33 neonates delivered by COVID-19-positive mothers, three had a SARS-CoV-2 infection. They indicated the symptoms were more related to prematurity than SARS-CoV-2. They observed leukocytosis and lymphocytopenia in a sick neonate [8]. During the early neonatal period, the blood count is influenced by many maternal and neonatal conditions, such as the presence of hypoglycemia, seizure, excess crying, infection, and metabolic conditions [9]. In a systematic review of 37 studies, among 302 neonates delivered to COVID-19-positive mothers, five babies had a complicated course, of which two neonates were SARS-CoV-2 infected [10]. Most neonates delivered to SARS-CoV-2-infected mothers are asymptomatic and need close clinical monitoring [11-13].

A systematic review of SARS-CoV-2 suggests that infected neonates’ abnormal leukocyte count and abnormal platelet count are found in 14% and 27% of cases, respectively [14]. In adult SARS-CoV-2-infected symptomatic patients, thrombocytopenia (20% to 55%) and lymphopenia (69.6% to 100%) are the most common abnormalities [15]. Leukopenia is the most common noted abnormality found in children, but normal leukocyte counts are found in most of the neonatal cases. Lymphopenia is rare in children compared to adults [16]. In a meta-analysis of 160 infants and neonates with COVID-19, the most common laboratory findings in infants were lymphocytosis (61%) and lymphopenia (16%) [17]. In our study, SARS-CoV-2-positive newborns had lower ANC and NLR than noninfected newborns, though similar effects were not observed when comparing asymptomatic neonates. Recent studies from low-income countries revealed that septic neonates have higher NLR than non-septic neonates [18,19]. However, the lower NLR in the SARS-CoV-2-infected newborns may be due to three septic neonates, compared to one septic neonate in the noninfected neonates. The leucopenia and lower ANC in symptomatic SARS-CoV-2-positive neonates may be explained by neonatal sepsis [20]. The trend of falling neutrophil counts in symptomatic SARS-CoV-2-positive newborns is likely due to the associated comorbidities or augmenting effect of SARS-CoV-2 infection. In a meta-analysis of 176 SARS-CoV-2-positive neonates, 97 had clinical symptoms related to SARS-CoV-2 infection, 14.4% had lymphopenia, 15.5% had elevated inflammatory markers (e.g., CRP and procalcitonin), and 4% had elevated liver enzymes [21]. We found that asymptomatic SARS-CoV-2-infected neonates had a normal CBC and had no significant difference in any hematological parameters than healthy (i.e., asymptomatic and free of SARS-CoV-2) neonates.

In this study, the biomarker of hepatic or renal dysfunction was not elevated in SARS-CoV-2-infected neonates. Multi-organ failure is less likely in neonatal SARS-CoV-2 infection, which may be more prevalent in the adult population due to comorbidities and different patterns of angiotensin-converting enzyme 2 (ACE2) expression [22]. The lower expression of ACE2 in neonates may explain the better prognosis in this population [23,24].

Neonates infected with SARS-CoV-2 had a high prevalence of prematurity (33.3%) and low birth weight (41.6%) compared to SARS-CoV-2-negative neonates (16.6% and 30.5%, respectively). Most (64.5%) COVID-19 mothers underwent cesarean section delivery.

Our study had a few important limitations. This is a single-center study with a small sample size. So, significantly lower TLC, ANC, and NLR in the SARS-CoV-2-positive newborns could not be taken as markers for severity or diagnosis purpose. Serial hematological and organ-specific biomarker changes were not included in the data analysis. Besides, we had not taken the clinical and laboratory test parameters, COVID-19 vaccine status, or COVID-19 antibody titre of COVID-19-positive mothers. Therefore, additional metacentric observational studies with larger sample sizes are needed to analyze the impact of SARS-CoV-2 on hematological changes in neonates.

## Conclusions

There is no significant difference in hematological and biochemical parameters between asymptomatic SARS-CoV-2-infected and -noninfected neonates. The blood count abnormalities found in SARS-CoV-2-infected neonates could be due to the overlapping effect of neonatal comorbidities. Asymptomatic SARS-CoV-2-infected neonates need close monitoring rather than a battery of investigational tests.
